# Integrative Metabolomics, Enzymatic Activity, and Gene Expression Analysis Provide Insights into the Metabolic Profile Differences between the Slow-Twitch Muscle and Fast-Twitch Muscle of *Pseudocaranx dentex*

**DOI:** 10.3390/ijms25116131

**Published:** 2024-06-01

**Authors:** Huan Wang, Busu Li, Ang Li, Changting An, Shufang Liu, Zhimeng Zhuang

**Affiliations:** 1 State Key Laboratory of Mariculture Biobreeding and Sustainable Goods, Yellow Sea Fisheries Research Institute, Chinese Academy of Fishery Sciences, Qingdao 266071, China; wanghuan@ysfri.ac.cn (H.W.); libusu1616@163.com (B.L.); liang@ysfri.ac.cn (A.L.); anct@ysfri.ac.cn (C.A.); zhuangzm@ysfri.ac.cn (Z.Z.); 2 Laboratory for Marine Fisheries Science and Food Production Processes, Qingdao Marine Science and Technology Center, Qingdao 266237, China

**Keywords:** *Pseudocaranx dentex*, slow-twitch muscle, fast-twitch muscle, untargeted metabolomics, energy metabolism, regulatory mechanism

## Abstract

The skeletal muscles of teleost fish encompass heterogeneous muscle types, termed slow-twitch muscle (SM) and fast-twitch muscle (FM), characterized by distinct morphological, anatomical, histological, biochemical, and physiological attributes, driving different swimming behaviors. Despite the central role of metabolism in regulating skeletal muscle types and functions, comprehensive metabolomics investigations focusing on the metabolic differences between these muscle types are lacking. To reveal the differences in metabolic characteristics between the SM and FM of teleost, we conducted an untargeted metabolomics analysis using *Pseudocaranx dentex* as a representative model and identified 411 differential metabolites (DFMs), of which 345 exhibited higher contents in SM and 66 in FM. KEGG enrichment analysis showed that these DFMs were enriched in the metabolic processes of lipids, amino acids, carbohydrates, purines, and vitamins, suggesting that there were significant differences between the SM and FM in multiple metabolic pathways, especially in the metabolism of energy substances. Furthermore, an integrative analysis of metabolite contents, enzymatic activity assays, and gene expression levels involved in ATP-PCr phosphate, anaerobic glycolysis, and aerobic oxidative energy systems was performed to explore the potential regulatory mechanisms of energy metabolism differences. The results unveiled a set of differential metabolites, enzymes, and genes between the SM and FM, providing compelling molecular evidence of the FM achieving a higher anaerobic energy supply capacity through the ATP-PCr phosphate and glycolysis energy systems, while the SM obtains greater energy supply capacity via aerobic oxidation. These findings significantly advance our understanding of the metabolic profiles and related regulatory mechanisms of skeletal muscles, thereby expanding the knowledge of metabolic physiology and ecological adaptation in teleost fish.

## 1. Introduction

Skeletal muscle constitutes a substantial proportion of fish body mass (35~60%) [1]. Such a high proportion underscores skeletal muscle’s pivotal role in powering swimming movements and maintaining metabolic homeostasis, thereby crucially impacting fish survival [2,3]. According to anatomical distribution and function, the skeletal muscle of most fish can mainly be categorized into two distinct types: slow-twitch muscle (SM) and fast-twitch muscle (FM) [4]. SM, located in the narrow wedge-shaped area near the lateral line, features relatively small fiber diameters and appears red or dark in color. It support sustained, low-frequency tail beats, facilitating prolonged swimming [5]. In contrast, FM, lying in the deep layers of the trunk, constitutes the bulk of the skeletal muscle mass. Characterized by larger fiber diameters and a white-to-off-white appearance [6], FM enables rapid, explosive movements essential for activities such as predation and escape [5].

Researchers have long endeavored to unravel the evolutionary adaptations that have shaped the distinct structural components and metabolic outputs of SM and FM, which reflect specificity their in functions. Histological studies have revealed differences in sarcomere lengths, Z-line widths, and densities of mitochondria and capillaries between the two muscle types [7,8,9,10]. Moreover, biochemical analyses have identified a wide disparity in myosin, the primary determinant of contractile properties, including differences in ATPase activity, actin-binding ability, filament formation, and structural stability [9,11]. Additionally, some studies have noted that SM contains higher amounts of lipid droplets, palmitic acid, and oleic acid [7,12], along with elevated levels of oxidation capacity [13] and lower pH [14]. Molecular biological investigations have delved into the regulatory mechanisms underlying these differences at epigenetic, transcriptional, and post-transcriptional levels, highlighting significant variations in the expression of proteins and genes associated with contraction, metabolism, and ion homeostasis pathways [15,16,17,18]. Although previous research has enriched our understanding of the physiological and biochemical distinctions between SM and FM, it has often focused on the transcript, protein, and phenotypic levels, leaving gaps between these areas. Metabolomics, which involves the characterization of changes in metabolites—the end products of gene expression—provides a physiological snapshot and allows for mapping the differences among cells, tissues, organs, or organisms resulting from an underlying trait [19]. These metabolic differences can elucidate functional relationships between genetic variations and biochemical phenotypes, which could enhance our understanding of key biochemical pathways. Unlike the species-specific differences observed at genomic, transcriptomic, and proteomic levels due to unique DNA sequences, metabolite structure and function are conserved across taxa, with their roles in specific metabolic processes being shared [20]. Therefore, a systematic examination of the metabolome is warranted to elucidate the metabolic patterns underlying muscle type differences. Skeletal muscle relies heavily on energy supply, and in fishes, three well-regulated pathways—the adenosine triphosphate–phosphocreatine (ATP-PCr) phosphate energy system, anaerobic glycolysis energy system, and aerobic oxidative energy system—have evolved to continuously replenish ATP and meet diverse energy demands [21]. It is widely acknowledged that SM primarily engages in aerobic metabolism, i.e., the aerobic oxidative energy system, to support sustained activities, while FM relies on anaerobic pathways, including ATP-PCr phosphate and glycolysis energy systems, to rapidly generate energy for burst activities. This difference is mainly discerned through measurements and comparisons of energy metabolism substrates such as lipids, mitochondria, capillaries, and glycogen [22], as well as the activities of some energy metabolism enzymes [23]. Since all three energy systems mentioned above involve a series of enzymatic reaction processes, the activities of enzymes can accurately and intuitively reflect the differences in metabolic capacity, and the analysis of gene expression profiles can provide insights into potential molecular regulatory mechanisms for metabolic pathways. Therefore, an integrated analysis of gene expression profiles, enzyme activities, and metabolite contents is expected to comprehensively reveal the regulatory mechanisms underlying the differences in energy metabolism between SM and FM.

The white trevally (*Pseudocaranx dentex*), a member of the Carangidae family, has garnered significant attention in the global mariculture industry due to its large size, rapid growth, disease resistance, and economic value [24,25]. As a pelagic migratory species, *P. dentex* exhibits a higher proportion of SM with increased mitochondria and capillary densities, enabling sustained swimming during oceanic migrations [26]. The skeletal muscle structure of *P. dentex* perfectly meets the needs for long-distance swimming, but little is known about its metabolic profiles and regulatory mechanisms, which has become a limitation for large-scale aquaculture efforts. Therefore, this study aims to investigate the global metabolic characteristics of SM and FM in *P. dentex,* using metabolomics analysis, and elucidates the upstream molecular regulatory mechanisms influencing differential energy metabolic pathways by integrating metabolite contents, enzymatic activity assays, and gene expression analysis. The results will provide a deep insight into the metabolic mechanisms of skeletal muscle in teleost.

## 2. Results

### 2.1. The Differences in Metabolite Profile between the SM and FM of P. dentex

#### 2.1.1. Overall Metabolic Profile of Muscles

SM and FM samples obtained from adult *P. dentex* (*n* = 6) were used for metabolome and subsequent analysis. A total of 701 metabolites were successfully identified via ultra-high-performance liquid chromatography coupled to tandem mass spectrometry (UHPLC-MS/MS) analysis, comprising 413 metabolites in positive-ion mode and 288 metabolites in negative-ion mode (Table S1). These metabolites encompassed a diverse range of compound classes, including 84 lipids and lipid-like molecules; 61 organic acids and derivatives; 37 organoheterocyclic compounds; 27 organic oxygen compounds; 23 nucleosides, nucleotides, and analogues; 11 benzenoids; 7 phenylpropanoids and polyketides; 6 organic nitrogen compounds; 1 alkaloid and derivatives; and 444 compounds yet to be definitively classified.

#### 2.1.2. Differences in Metabolite Profiles between SM and FM

**Figure 1:**
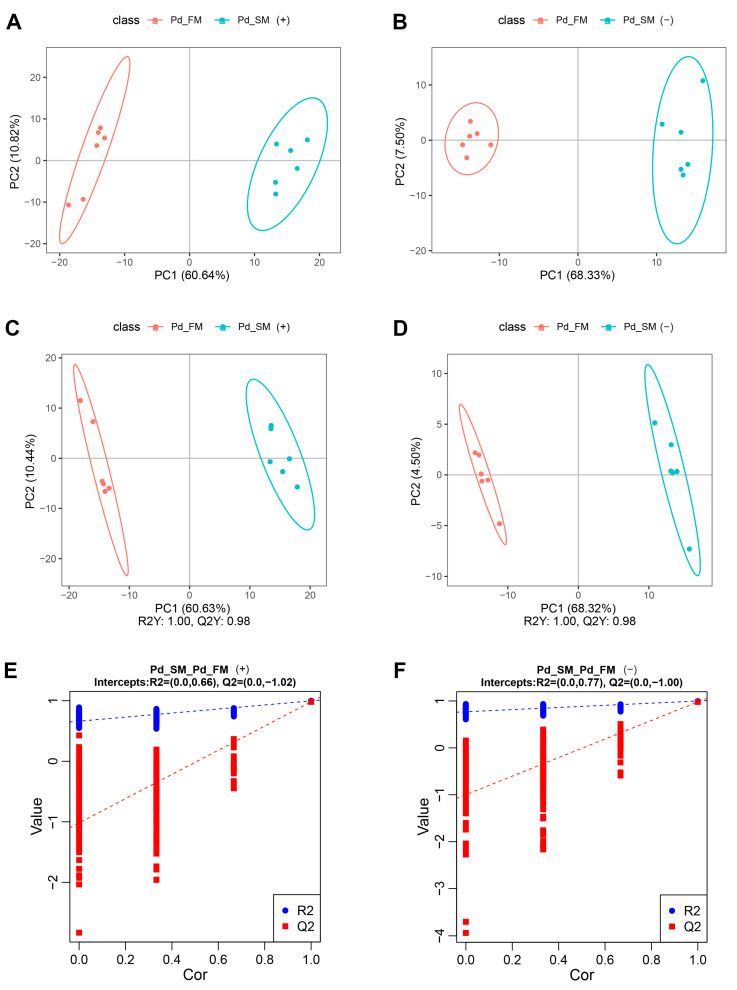
Multivariate statistical analysis of all samples for untargeted metabolomics in positive-ion mode (**A**,**C**,**E**) and negative-ion mode (**B**,**D**,**F**). (**A**,**B**) PCA score plots of the first two components between SM and FM; (**C**,**D**) PLS-DA score plots of the first two components between SM and FM; (**E**,**F**) permutation test from PLS-DA models.

Principal component analysis (PCA) showed that all the samples fell within the 95% confidence interval, with the first principal component effectively segregating the samples into two distinct groups along the *x*-axis (Figure 1A,B). Partial least squares discrimination analysis (PLS-DA) further illustrated a clear separation between the SM and FM samples in both positive-ion mode (R^2^Y (cumulative (CUM)) = 1.00, Q^2^Y (CUM) = 0.98) (Figure 1C) and negative-ion mode (R^2^Y (CUM) = 1.00, Q^2^Y (CUM) = 0.98) (Figure 1D). Permutation analysis (positive-ion: Q2 intercept = −1.02, negative-ion: Q2 intercept = −1.00) (Figure 1E,F) confirmed the robustness of the PLS-DA model, demonstrating its excellent interpretive and predictive capabilities without overfitting. Overall, these results underscore the substantial differences in metabolite profiles between the SM and FM of *P. dentex*.

**Figure 2:**
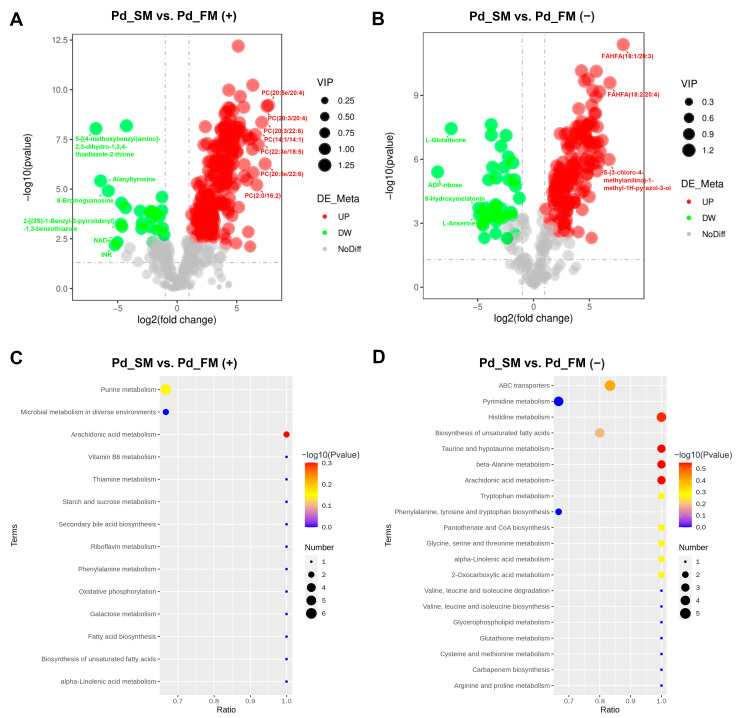
The expression profiles and functional enrichment analysis of DFMs between SM and FM in positive-ion mode (**A**,**C**) and negative-ion mode (**B**,**D**). (**A**,**B**) Volcano plots for the DFMs features; (**C**,**D**) KEGG pathway analysis for the DFMs.

Based on the cutoff criteria (variable importance of projection (VIP) > 1.0, *p* < 0.05, and fold change (FC) ≥ 1.5 or FC ≤ 0.667), a total of 411 differential metabolites (DFMs) were identified between the SM and FM, with 226 in the positive-ion mode and 185 in the negative-ion mode. Among these, 345 DFMs exhibited significant upregulation in the SM (200 in positive-ion mode and 145 in negative-ion mode), with almost all of the top 10 being lipids and lipid-like molecules (Figure 2A,B). In addition, 58 of the 60 DFMs belonging to the lipids and lipid-like molecules superclass were expressed at higher levels in the SM.

Conversely, 66 DFMs were significantly upregulated in the FM (26 in positive-ion mode and 40 in negative-ion mode), with the top 10 metabolites exhibiting diverse characteristics, including nucleosides, nucleotides, organoheterocyclic compounds, and organic acids and derivatives (Figure 2A,B). Moreover, among 28 DFMs that belong to the organic acids and derivatives, 18 were expressed at higher levels in the FM, including L-histidine and creatine.

KEGG enrichment analysis revealed a total of 14 and 27 functional pathways enriched by DFMs in the positive and negative-ion modes, respectively. These pathways primarily included the metabolism of lipids (e.g., arachidonic acid and alpha-linolenic acid), amino acids (e.g., beta-alanine, taurine and hypotaurine, and histidine et al.), ABC transporters, purine, carbohydrate, and vitamins (Figure 2C,D).

### 2.2. The Differential Contents of Metabolites Related to Energy Metabolism between SM and FM of P. dentex

To explore the differences in energy metabolism between SM and FM, we conducted a comparative analysis using metabolomics data on key metabolites involved in ATP-PCr, anaerobic glycolysis, and aerobic oxidative energy metabolism systems.

**Figure 3:**
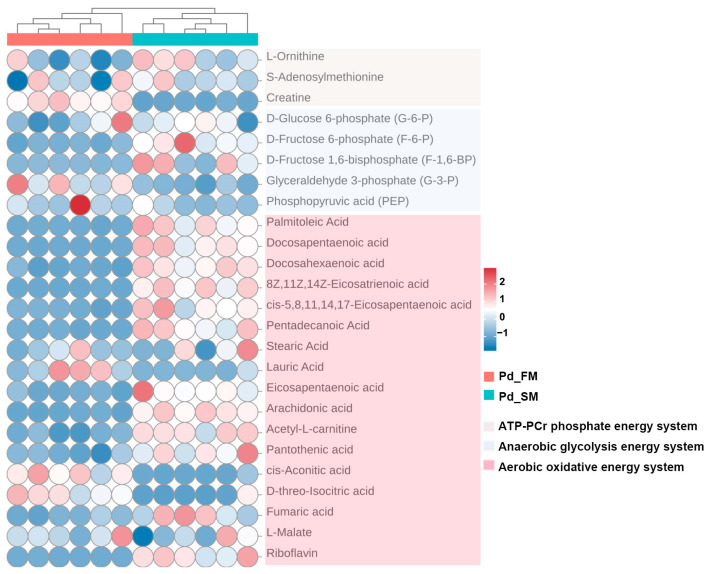
Heatmap of key metabolites related to ATP-PCr, anaerobic glycolysis, and aerobic oxidative energy metabolism systems, identified using untargeted metabolomics between the SM and FM of *P. dentex*. The clustering analysis at the top showed that the similar metabolite profiles in the six Pd_FM samples demonstrated good biological repeatability. The same pattern is also observed for Pd_SM samples.

As shown in Figure 3, creatine, a crucial component of the ATP-PCr energy metabolism system, was found to have a higher content in FM.

Within the anaerobic glycolysis energy system, glyceraldehyde 3-phosphate (G-3-P) and phosphoenolpyruvate (PEP) were found to be more abundant in the FM, while the SM exhibited increased levels of D-fructose 6-phosphate (F-6-P) and D-fructose 1, 6-bisphosphate (F-1,6-BP) (Figure 3).

Furthermore, analysis of the aerobic oxidative energy system indicated elevated levels of various fatty acids substrates involved in β oxidation (palmitoleic acid, docosapentaenoic acid, docosahexaenoic acid, 8Z,11Z,14Z-eicosatrienoic acid, cis-5,8,11,14,17-eicosapentaenoic acid, pentadecanoic acid, lauric acid, eicosapentaenoic acid, and arachidonic acid), along with an increased abundance of acetyl-L-carnitine (the derivative of carnitine), pantothenic acid (the precursor of acetyl-CoA), intermediate metabolite fumarate, and riboflavin (the precursor of hydrogen delivery substance (FAD)) in SM. Conversely, the FM exhibited higher concentrations of cis-aconitic acid and D-threo-isocitric acid (Figure 3).

Therefore, the above results identified multiple distinct metabolites across the three energy metabolic systems in SM and FM, indicating significant differences in energy metabolism at the metabolite level. SM appears to have a higher capacity for aerobic oxidation, whereas FM exhibits greater ATP-PCr energy supply capacity. However, the glycolytic energy supply capacity cannot be accurately assessed based on metabolite levels alone.

### 2.3. The Differential Activities of Enzymes Related to Energy Metabolism between the SM and FM of P. dentex

**Figure 4:**
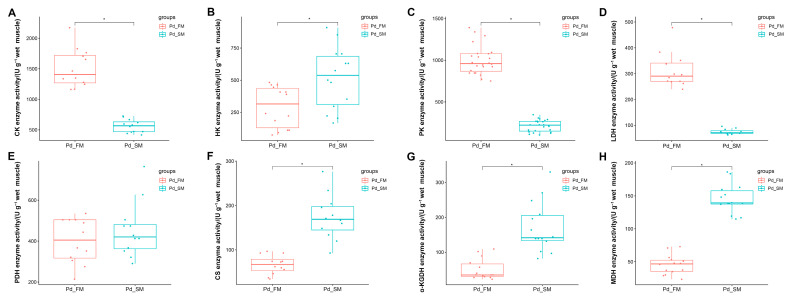
Differential analysis of enzyme activities related to energy metabolism between the SM and FM of *P. dentex* (*n* = 6). * indicates significant differences (*p* < 0.05) between the SM and FM. (**A**) Enzyme related to ATP-PCr phosphate energy system, CK activity, was significantly greater in FM than in SM. (**B**–**E**) Enzymes involved in anaerobic glycolysis energy system, (**B**) HK activity was significantly greater in SM than in FM; (**C**) PK activity was significantly greater in FM than in SM; (**D**) LDH activity was significantly greater in FM than in SM; (**E**) PDH activity did not differ between FM and SM. (**F**–**H**) Enzymes involved in aerobic oxidative energy system, (**F**) CS activity was significantly greater in SM than in FM; (**G**) α-KGDH activity was significantly greater in SM than in FM; (**H**) MDH activity was significantly greater in SM than in FM.

In the ATP-PCr phosphate energy system, the FM exhibited significantly higher creatine kinase activity (CK) compared to the SM (*p* < 0.05) (Figure 4A).

Within the anaerobic glycolysis energy system, the activities of pyruvate kinase (PK) and lactate dehydrogenase (LDH) were markedly elevated in the FM compared to the SM (*p* < 0.05) (Figure 4C,D), while the SM displayed a significantly higher hexokinase (HK) activity than the FM (*p* < 0.05) (Figure 4B). Pyruvate dehydrogenase (PDH) activity did not differ significantly between the SM and the FM (Figure 4E).

In the aerobic oxidative energy system, the activities of citrate synthase (CS), α-ketoglutarate dehydrogenase (α-KGDH), and malic dehydrogenase (MDH) were all significantly higher in the SM compared to the FM (Figure 4F–H).

These results further underscore the distinctions in energy metabolism between SM and FM at the enzyme activity level. Specifically, SM may possess a greater capacity for aerobic oxidation, whereas FM exhibits a higher ATP-PCr energy supply capacity. Additionally, FM might demonstrate an elevated glycolytic energy supply capacity, as suggested by enzyme levels.

### 2.4. The Differential Expression Profiles of Genes Related to Energy Metabolism between the SM and FM of P. dentex

**Figure 5:**
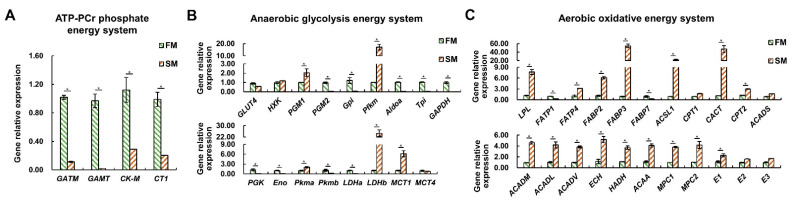
Differential expression profiles of genes related to energy metabolism between the SM and FM of *P. dentex* (*n* = 6). (**A**) Genes related to ATP-PCr phosphate energy system. (**B**) Genes involved in anaerobic glycolysis energy system. (**C**) Genes participating in aerobic oxidative energy system. The relative expression levels of genes were normalized to *β-Actin* by performing three technical replicates. * indicates significant differences (*p* < 0.05) between the SM and FM.

In the context of the ATP-PCr phosphate energy system, mRNA expression levels of four key gene—glycine amidinotransferase (*GATM*), guanidinoacetate N-methyltransferase (*GAMT*), muscle-type creatine kinase (*CK-M*), and Cr transporter (*CT1*)—were significantly higher in the FM than in the SM (*p* < 0.05) (Figure 5A), indicating that the FM possesses stronger Cr synthesis and uptake abilities, which can support the ATP-PCr energy system.

In the anaerobic glycolysis energy system, transcripts of phosphoglycerate mutase 2 (*PGM2*), glucose-6-phosphate isomerase (*Gpi*), aldolase (*Aldoa*), triose phosphate isomerase (*Tpi*), glyceraldehyde 3-phosphate dehydrogenase (*GAPDH*), phosphoglycerate kinase (*PGK*), enolase (*Eno*), pyruvate kinase b (*Pkmb*), and lactate dehydrogenase a (*LDHa*) were significantly higher in the FM compared to the SM (*p* < 0.05) (Figure 5B). Conversely, the mRNA expression levels of phosphoglycerate mutase 1 (*PGM1*), muscle-type phosphofructokinase (*Pfkm*), pyruvate kinase a (*Pkma*), lactate dehydrogenase b (*LDHb*), and monocarboxylate transporter 1 (*MCT1*) were higher in the SM than in the FM (*p* < 0.05) (Figure 5B). These results suggest that FM has a stronger anaerobic glycolysis capacity than SM, as is mainly reflected in the last seven steps at the transcriptional level.

In the aerobic oxidative energy system, mRNA levels of lipoprotein lipase (*LPL*), fatty acid transport protein 4 (*FATP4*), fatty acid binding protein 2 (*FABP2*) and 3 (*FABP3*), acyl-coenzyme A synthetase 1 (*ACSL1*), carnitine–acylcarnitine translocase (*CACT*), carnitine palmitoyltransferase II (*CPT2*), acyl-CoA dehydrogenase medium chain (*ACADM*), acyl-CoA dehydrogenase long chain (*ACADL*), acyl-CoA dehydrogenase very long chain (*ACADV*), enoyl-CoA hydratase (*ECH*), 3-hydroxyacyl-CoA dehydrogenase (*HADH*), 3-ketoacyl-CoA thiolase (*ACAA*), pyruvate carrier 1 (*MPC1*) and 2 (*MPC2*), and pyruvate dehydrogenase (*E1*) were significantly higher in the SM compared to the FM (*p* < 0.05) (Figure 5C). Only fatty acid transport protein 1 (*FATP1*) and fatty acid binding protein 7 (*FABP7*) were higher expressed in the FM compared to the SM (*p* < 0.05) (Figure 5C). These results unquestionably support the greater aerobic oxidation capacity of SM than FM.

Overall, the gene expression data consistently corroborated the findings regarding metabolite content and enzyme activity, indicating that SM exhibits a preference for aerobic metabolism compared to FM, which relies heavily on anaerobic metabolism. Furthermore, our analysis suggests that transcriptional regulation may not be the primary mechanism that determines the enzyme activity of HK.

## 3. Discussion

Metabolites, as pivotal phenotypic constituents, accurately depict the physiological characteristics of skeletal muscle [27]. Untargeted metabolomics, also known as discovery metabolomics, serves as a robust method capable of the large-scale identification and quantification of metabolites, thus providing valuable insights into skeletal muscle physiology. In the present study, we employed UHPLC-MS/MS untargeted metabolomics to explore metabolite profiles and compare differences in metabolic pathways between the SM and the FM of *P. dentex*. Our analysis unveiled a significantly larger number of identified metabolites compared to previous studies that focused solely on the dorsal FM of *Ctenopharyngodon idella* and *Megalobrama amblycephala* [28,29], indicating the comprehensiveness of the metabolite profiles identified in this study.

Fish skeletal muscle plays crucial roles in various physiological processes, including movement, body support, and metabolic homeostasis. In this study, significant functional differences between SM and FM were reflected by the DFMs enriched in many metabolic pathways, including lipids (e.g., arachidonic acid and alpha-linolenic acid), amino acids (e.g., beta-alanine, taurine, hypotaurine, and histidine et al.), ABC transporters, purine, carbohydrate, and vitamins. Lipid and carbohydrate metabolism deliver pivotal fuels that provide energy for various skeletal muscle functions [30]. Vitamins act as essential cofactors or coenzymes in metabolic reactions involving carbohydrates, proteins, and lipids [31]. Beta-alanine and histidine serve as precursors of carnosine and other histidine-containing dipeptides (HCDs), which aid in buffering intramuscular pH, enhancing anaerobic exercise performance, and improving anoxia tolerance in FM [32]. In SM, taurine contributes to pH buffering within the alkaline pH range of 7.0–8.0 [33] and regulates fatty acid aerobic oxidation [34]. ABC transporters utilize ATP hydrolysis to pump compounds across the membrane [35], and purine metabolites (ATP, ADP, and adenosine et al.) act as signaling molecules that engage G protein-coupled or ligand-gated ion channel receptors [36]. These differential metabolites and pathways shed light on the diverse metabolic functions between SM and FM.

**Figure 6:**
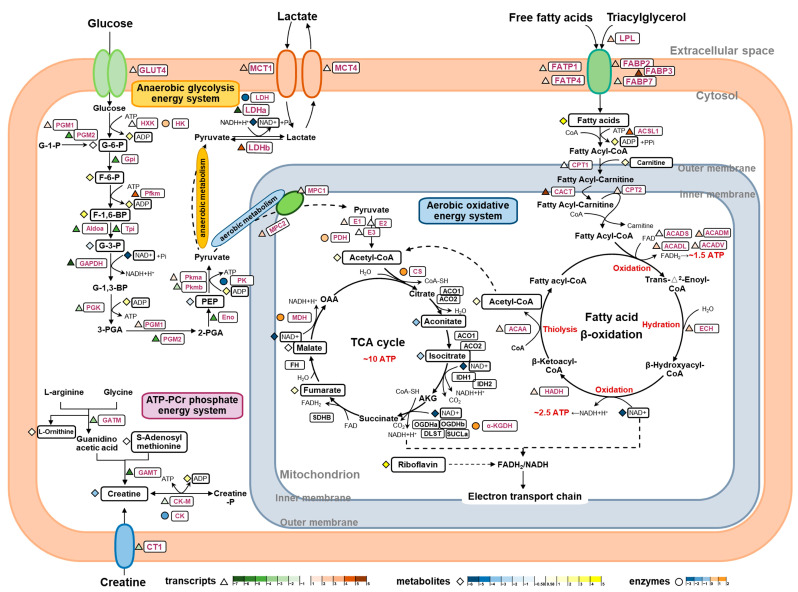
An integrated analysis of metabolite profiles, enzyme activities, and gene expression levels elucidate the molecular pathways regulating the differences in the three energy metabolism systems between the SM and FM of *P. dentex*. The color of triangles represent the log2 (fold change) of the genes between the SM and FM, with red and green representing higher expression in the SM and FM, respectively. The color of diamonds represent the log2 (fold change) of the metabolites between the SM and FM, with yellow and blue representing higher expression in the SM and FM, respectively. The color of circles represent the log2 (fold change) of the enzyme activities between the SM and FM, with orange and cyan representing higher expression in the SM and FM, respectively.

As SM and FM are specialized to support two distinct swimming modes, continuous and burst swimming, their energy supply processes differ accordingly. The results from the metabolomics, enzyme assays, and gene expression analysis indicate specific differences in the three energy metabolism pathways responsible for ATP replenishment between the SM and FM of *P. dentex*, as illustrated in Figure 6.

The ATP-PCr phosphate energy system generates large amounts of energy for muscles during the initial 1 to 15 s of high intensity activity, but it last for a short time. The enzymes GATM and GAMT are responsible for creatine (Cr) synthesis from L-arginine, S-adenosyl-methionine, and glycine [37], and CT1 facilitates the transport of Cr from exogenous sources into muscle cells [38]. *GATM*, *GAMT*, and *CT1* were all significantly more expressed in the FM compared to the SM, consistent with the higher content of Cr in FM, suggesting that FM possesses a stronger capacity for Cr synthesis and uptake. PCr/Cr homeostasis is mainly regulated by the phosphorylation and de-phosphorylation of CK-M in skeletal muscle [39]. To a certain extent, CK-M represents the ATP regeneration capacity by P(Cr) [40]. In this study, both the gene transcriptional level and enzyme activity of CK were higher in FM, indicating that FM has a greater ability to replenish ATP through the ATP-PCr system in a short period of time, thus quickly providing energy support for its short-term explosive movement.

The energy produced by the anaerobic glycolysis system, without oxygen consumption, enables short bursts of high-intensity swimming by rapidly reaching near-maximal rates and sustaining them for several seconds. Traditionally, with its poor oxygen supply and low mitochondrial content, FM was considered to be more adapted for anaerobiosis [41]. In *P. dentex*, this was supported by the higher expression levels of a range of components involved in the last seven steps in the glycolytic pathway. Firstly, higher contents of G-3-P were found in FM, which isomerized from F-1,6-BP by elevated transcript levels of *Aldoa* and *Tpi*. Subsequently, G-3-P is oxidized, transacylated, mutated, and dehydrated into more PEP, regulated by higher mRNA levels of *GAPDH*, *PGK*, *PGM2*, and *Eno*, eventually converting into pyruvate and lactate by the catalysis of PK and LDH at markedly elevated enzyme activities and transcript levels in the FM [42]. The above differences in these intermediates, enzyme activities, and gene expressions suggest that the FM of *P. dentex* has a higher anaerobic glycolysis capacity to provide higher power outputs for its burst movement by elevating the last seven steps’ capacity. Additionally, we found that the SM exhibited almost the same G-6-P content as the FM and was capable of phosphorylation to form more F-6-P, F-1, and 6-BP, possibly regulated by higher mRNA levels of *PGM1* and *Pfkm*. Meanwhile, HK enzyme activity was found to be even higher in the SM. These findings suggest that SM also harbors a degree of anaerobic glycolytic capacity. However, this capacity may diminish in later stages due to reductions in the expression levels and activities of rate-limiting enzymes.

The aerobic energy system requires the oxidation of carbohydrates and lipids in the presence of sufficient oxygen to offer substantial energy, albeit at a somewhat constrained in its delivery rate. In teleost, superficial SM sustains prolonged swimming activity, primarily fueled by the aerobic oxidative energy system attributed to its extensive vascular supply, high mitochondrial and myoglobin content, and elevated oxygen utilization rate [43]. In this study, increased levels of pantothenic acid (a precursor of Acetyl-CoA, the initiator of the TCA cycle), riboflavin (a precursor of FAD, which participates in the electron transport chain as hydrogen-delivering substance), and fumarate and malate (two intermediates of the TCA cycle), coupled with heightened enzyme activities of CS, α-KGDH, and MDH, underscore the enhanced oxidative energy supply capacity of the SM in *P. dentex*. Lipids, as the primary energy substrate for aerobic metabolism, are pivotal fuel sources during sustained swimming in fish [44,45]. Upregulated transcription levels of *LPL*, *FABP*, and *FATP* promote the release and transport of more fatty acids into SM for uptake and utilization. Subsequently, more fatty acids are activated and transported into mitochondria, corresponding to increased transcripts of *ACSL1*, *CPT1*, *CACT*, and *CPT2* along with a high abundance of carnitine derivatives [46]. Moreover, increased mRNA levels of four enzymes, *ACAD*, *ECH*, *HADH*, and *ACAA*, involved in fatty acids *β*-oxidation, could augment the respective enzymatic contents and enhance lipid oxidation in the SM, thereby bolstering aerobic capacity and providing more energy.

Another contributor to aerobic oxidation is pyruvate, the final product of carbohydrate glycolysis. Serving as the exclusive entry point for pyruvate into the mitochondrial matrix for subsequent participation in the TCA cycle and electron transport chain, the MPCs play a crucial role in coordinating glycolytic and mitochondrial activities, providing a key juncture for regulating cellular energy production and metabolism [47]. Within mitochondria, the oxidation of pyruvate by PDH yields acetyl-CoA, which can subsequently condense with oxaloacetate (OAA) to generate citrate, the initial substrate of the TCA cycle [48]. Upregulated expressions of *MPC1*, *MPC2*, and *E1* may facilitate the import of more pyruvate into the mitochondrion, thereby increasing acetyl-CoA production for aerobic metabolism in the SM. This supports the hypothesis that SM exhibits a higher capacity for aerobic pyruvate oxidation, sustaining prolonged swimming activity. Moreover, increased transcripts of *MCT1* and *LDHb* in SM facilitate the transfer of lactate from glycolytic to oxidative muscle cells, where it can be converted to pyruvate for oxidative utilization [49]. These findings demonstrate that SM may be more adept at oxidizing lactate generated by the anaerobic glycolysis of FM.

## 4. Materials and Methods

### 4.1. Sample Collection

SM and FM samples were obtained from six healthy adult specimens of *P. dentex*, with an average body length of 36.40 ± 1.04 cm and a body weight of 1411.06 ± 224.65 g. These specimens were randomly collected from Dalian Tianzheng Industrial Co., Ltd. (Dalian, Liaoning province, China) in November 2020. Before sampling, the living fish were anesthetized using MS-222 (Sigma Aldrich Chemie GmbH, Steinheim, Germany) to minimize suffering. The SM was collected from the zone beneath lateral line, while the FM was collected from the dorsal epaxial region, with any ambiguous fibers carefully removed. All samples were promptly preserved at −80 °C until further analysis.

### 4.2. Untargeted-Metabolomics Metabolite Profiling Analysis

#### 4.2.1. Metabolites Extraction

A weighed 100 mg frozen sample was ground to a fine powder using liquid nitrogen and then re-suspended in pre-chilled 80% methanol by vortex. Following a 5 min incubation on ice, the homogenate was centrifuged at 15,000× *g* for 20 min at 4 °C. The resulting supernatant was collected and diluted with LC-MS-grade water. After another centrifugation step for 20 min at 15,000× *g* and 4 °C, the supernatant was collected, filtered and injected into the UHPLC-MS/MS analysis system. Quality control samples (QCs) were prepared by mixing equal volumes of the experimental samples.

#### 4.2.2. UHPLC-MS/MS Analysis

Metabolite separation and analysis were conducted using a Vanquish UHPLC system (ThermoFisher, Bremen, Germany) coupled with an Orbitrap Q Exactive^TM^ HF mass spectrometer (ThermoFisher, Bremen, Germany) at Novogene Co., Ltd. (Beijing, China). Samples were injected into a Hypesil Gold column (100 mm × 2.1 mm, 1.9 μm) at a flow rate of 0.2 mL/min, employing a 17 min linear gradient. The eluents consisted of eluent A (0.1% formic acid in water) and eluent B (methanol) for positive polarity mode and eluent A (5 mM ammonium acetate, pH 9.0) and eluent B (methanol) for negative polarity mode. The elution condition was programmed as follows: 0–1.5 min, 2% B; 1.5–3 min, 2–100% B; 3–10 min, 100% B; 10–10.1 min, 100–2% B; and 10.1–12 min, 2% B. The Q Exactive^TM^ HF mass spectrometer operated in the positive/negative polarity modes with a spray voltage of 3.5 kV, capillary temperature of 320 °C, sheath gas flow rate of 35 psi, aux gas flow rate of 10 L/min, S-lens RF level of 60, and aux gas heater temperature of 350 °C.

The QCs were inserted into the sample queue at intervals of every 10 samples throughout the analytical run to monitor and evaluate the stability of the system and the reliability of the experimental data.

#### 4.2.3. Data Preprocessing and Metabolite Identification

Raw data files generated by using UHPLC-MS/MS were preprocessed using Compound Discoverer 3.1 (CD3.1, ThermoFisher, Bremen, Germany) through peak alignment, peak picking, and quantitation for each metabolite. The main parameters were configured as follows: retention time tolerance of 0.2 min, actual mass tolerance of 5 ppm, signal intensity tolerance of 30%, signal/noise ratio of 3, and minimum peak intensity of 50,000. Following normalization to the total spectral intensity, the normalized peak intensities were utilized for molecular formula prediction based on additive ions, molecular ion peaks, and fragment ions. Subsequently, the mzCloud (https://www.mzcloud.org/, accessed on 12 September 2023), mzVault 1.0sp1 (Thermo Fischer Scientific, Waltham, MA, USA), and MassList databases (Thermo Fischer Scientific, Waltham, MA, USA) were used for matching peaks to obtain accurate qualitative and relative quantitative results.

Statistical analyses were performed using the R package (R-3.4.3 version), Python (Python 2.7.6 version), and CentOS (CentOS release 6.6). When data were not normally distributed, the area normalization method was used for normal transformation.

Metabolites were annotated using the KEGG database (https://www.genome.jp/kegg/pathway.html, accessed on 5 October 2023), HMDB database (https://hmdb.ca/metabolites, accessed on 18 October 2023), and LIPIDMaps database (http://www.lipidmaps.org/, accessed on 1 November 2023).

#### 4.2.4. Multivariate Data Analysis and Univariate Statistical Analysis

The data matrix was imported into an R package, metaX [50], for multivariate statistical analysis, which included PCA, PLS-DA, and VIP. Univariate analysis (*t*-test) was employed to evaluate the statistical significance (*p*-value). Metabolites meeting the following criteria were identified as DFMs: VIP > 1.0, *p*-value < 0.05, and FC ≥ 1.5 or FC ≤ 0.667. Volcano plots were constructed to filter metabolites of interest based on log2(FC) and −log10(*p*-value) by using ggplot2. The main metabolic pathways and signal transduction processes of DFMs were annotated using the KEGG database (https://www.genome.jp/kegg/pathway.html, accessed on 12 November 2023).

To further explore the differences in energy metabolism between the SM and FM, a heatmap was constructed using the pheatmap package to visualize the key metabolites related to ATP-PCr, anaerobic glycolysis, and aerobic oxidative energy metabolism systems, as identified by untargeted metabolomics.

### 4.3. Energy Metabolite Enzymatic Activity Assays

Eight key enzymes that were involved in the three energy metabolism systems, ATP-PCr phosphate energy system, anaerobic glycolysis energy system, and aerobic oxidative energy system, were assayed and compared for their activities between the SM and FM.

The ATP-PCr phosphate energy system, CK (E.C.2.7.3.2), is pivotal for catalyzing the reversible phosphorylation reaction between creatine and ATP in, playing a crucial role in energy transfer, muscle contraction, and ATP regeneration. Its activity was assessed by measuring ATP formation at 340 nm in 0.05 g samples using the Creatine Kinase Activity Assay Kit (Solarbio BC1145, China), following the manufacturer’s instructions.

The anaerobic glycolysis energy system, HK (E.C.2.7.1.1), the primary enzyme initiating glucose breakdown, was assayed by monitoring NADPH formation at 340 nm in 0.1 g samples with the Hexokinase Activity Assay Kit (BC0745, Solarbio, Beijing, China), following the manufacturer’s guidance. PK (E.C.2.7.1.40), which catalyzes the conversion of phosphoenolpyruvate to pyruvate and is a key indicator of glycolytic potential activity, was determined by assessing the decline rate of NADH in 0.05 g samples at 340 nm using the Pyruvate Kinase Activity Assay Kit (BC0545, Solarbio, Beijing, China), per the manufacturer’s protocol. LDH (EC 1.1.1.27), the terminal enzyme in anaerobic glycolysis, was evaluated by measuring pyruvate formation at 450 nm in 0.1 g samples with the Lactate Dehydrogenase Activity Assay Kit (BC0685, Solarbio, Beijing, China), according to the manufacturer’s instructions. PDH (E.C.4.1.1.1), a rate-limiting enzyme for oxidative decarboxylation of pyruvate and linking glycolysis to the TCA cycle, was assessed by quantifying 2,6-DCPIP absorption at 605 nm in 0.1 g samples using the Pyruvate Dehydrogenase Activity Assay Kit (BC0385, Solarbio, Beijing, China), according to the manufacturer’s instructions.

The aerobic oxidative energy system, CS (E.C.4.1.3.7), which catalyzes the initial step of the TCA cycle, was assessed by measuring coenzyme A formation at 412 nm in 0.2 g samples using the Citrate Synthase Activity Assay Kit (BC1065, Solarbio, Beijing, China), following the manufacturer’s instructions. α-KGDH (E.C.1.2.4.2), another crucial enzyme in TCA cycle regulation, was determined by measuring NADH formation in 0.2 g samples at 340 nm with the α-Ketoglutarate Dehydrogenase Activity Assay Kit (BC0715, Solarbio, Beijing, China), according to the manufacturer’s protocol. MDH (E.C.1.1.1.37), an enzyme integral to the TCA cycle and to maintaining redox balance between mitochondria and cytoplasm, was measured by monitoring malic acid formation in 0.2 g samples at 340 nm using the Malate Dehydrogenase Activity Assay Kit (BC1045, Solarbio, Beijing, China), as per the manufacturer’s instructions.

### 4.4. Gene Expression Analysis

The expression levels of 4 genes involved in the ATP-PCr phosphate energy system, 17 genes involved in the anaerobic glycolysis energy system, and 22 genes involved in the aerobic oxidative energy system were quantitatively compared between the SM and FM. The detailed gene list and primer sequences used in this study are shown in Table S2. RNA extraction from the samples was carried out using RNAiso Plus (Takara, Beijing, China), following the manufacturer’s instructions. For each sample, 1.0 μg of total RNA was reverse-transcribed using the PrimeScipt^TM^ RT reagent kit with gDNA Eraser (Takara, Shiga, Japan). Gene expression levels were quantified using TB Green Premix DimerEraser (Takara, Shiga, Japan) in a 7500 Fast real-time PCR system (Applied Biosystems, Foster, CA, USA). *β-Actin* was employed as the internal reference gene for normalization, and relative gene expression levels were determined using the 2^−∆∆Ct^ method.

### 4.5. Statistical Analysis

Statistical analyses were performed using SPSS 23.0 software (SPSS Inc., Chicago, IL, USA). One-way ANOVA was utilized to compare significant differences between different muscle types. The Student’s two-tailed *t*-test was used to identify components that differed significantly between the SM and FM. Statistical significance was set at *p* < 0.05.

## 5. Conclusions

In summary, this study provides comprehensive insights into the metabolic characteristics and regulatory mechanisms underlying the differential energy metabolism between SM and FM in *P. dentex*. There were significant differences between the SM and FM in multiple metabolic pathways, especially in the metabolism of energy substances according to untargeted metabolomics analysis. To satisfy the energy demand of explosive swimming, FM exhibits a stronger anaerobic energy supply capacity, which is achieved by increasing the synthesis, uptake, and phosphorylation of Cr in the ATP-PCr energy system, and by elevating the last seven steps in the anaerobic glycolysis energy system. SM possesses a stronger energy supply capacity through aerobic oxidation, which is supported by higher contents and oxidation utilization rates of fatty acids and pyruvate. Overall, this study may help to advance our understanding of the metabolic profiles and regulatory mechanisms of skeletal muscles, thereby expanding the knowledge of metabolic physiology and ecological adaptation in teleost fish.

## Data Availability

Data are contained within the article and Supplementary Materials.

## Supplementary Materials

The following supporting information can be downloaded at: https://www.mdpi.com/article/10.3390/ijms25116131/s1.
